# Emerging Trends in Dissolving-Microneedle Technology for Antimicrobial Skin-Infection Therapies

**DOI:** 10.3390/pharmaceutics16091188

**Published:** 2024-09-08

**Authors:** Rui Luo, Huihui Xu, Qiaoni Lin, Jiaying Chi, Tingzhi Liu, Bingrui Jin, Jiayu Ou, Zejun Xu, Tingting Peng, Guilan Quan, Chao Lu

**Affiliations:** 1 State Key Laboratory of Bioactive Molecules and Druggability Assessment, Department of Pharmacy, Jinan University, Guangzhou 511436, China; 2 Department of Pharmacy, College of Pharmacy, Jinan University, Guangzhou 511436, China; 3 Department of Chemistry, University of South Florida, Tampa, FL 33620, USA

**Keywords:** dissolving microneedles, antimicrobial therapy, skin infection, drug delivery systems, druggability

## Abstract

Skin and soft-tissue infections require significant consideration because of their prolonged treatment duration and propensity to rapidly progress, resulting in severe complications. The primary challenge in their treatment stems from the involvement of drug-resistant microorganisms that can form impermeable biofilms, as well as the possibility of infection extending deep into tissues, thereby complicating drug delivery. Dissolving microneedle patches are an innovative transdermal drug-delivery system that effectively enhances drug penetration through the stratum corneum barrier, thereby increasing drug concentration at the site of infection. They offer highly efficient, safe, and patient-friendly alternatives to conventional topical formulations. This comprehensive review focuses on recent advances and emerging trends in dissolving-microneedle technology for antimicrobial skin-infection therapy. Conventional antibiotic microneedles are compared with those based on emerging antimicrobial agents, such as quorum-sensing inhibitors, antimicrobial peptides, and antimicrobial-matrix materials. The review also highlights the potential of innovative microneedles incorporating chemodynamic, nanoenzyme antimicrobial, photodynamic, and photothermal antibacterial therapies. This review explores the advantages of various antimicrobial therapies and emphasizes the potential of their combined application to improve the efficacy of microneedles. Finally, this review analyzes the druggability of different antimicrobial microneedles and discusses possible future developments.

## 1. Introduction

Local infection refers to the process by which pathogenic bacteria invade an organism, colonize certain parts (including the skin, soft tissues, and internal organs), continuously grow and multiply, produce toxic substances, and constantly attack the organism. If a local infection is not promptly diagnosed and treated, pathogenic bacteria can easily enter the bloodstream through the sites of local infection and continue to grow and multiply in the body, releasing toxins that can cause systemic infections and even endanger life. Skin and soft-tissue infections (SSTIs) are the most common localized infections and are among the most prevalent bacterial infections affecting human health [1]. They primarily involve inflammatory diseases caused by pathogenic bacteria invading the skin, subcutaneous tissues, fascia, and muscles, including impetigo, ecthyma, folliculitis, furuncles, carbuncles, abscesses, trauma-related infections, decubital ulcers, and infections from human or animal bites. More severe SSTIs include cellulitis, erysipelas, and necrotizing fasciitis, which may lead to septic shock or other systemic symptoms of toxicity [2].

An SSTI may be owing to a single bacterial infection or a mixture of multiple bacteria. The most common pathogens that cause complicated SSTIs are *Staphylococcus aureus* (*S. aureus*) and *Streptococcus aureus*, followed by a variety of Gram-positive and Gram-negative aerobic bacteria [3]. The wide variety of bacterial species causing SSTIs and severe bacterial resistance has led to a continuous increase in prevalence, high recurrence rates, and difficulty in clinical treatment, resulting in a greater burden of healthcare costs on society [4]. Currently, SSTI has become one of the causes of death with the greatest clinical concern; for example, in a typical necrotizing soft-tissue infection, infected tissues develop fever, erythema, and swelling with rapid discoloration, as well as blisters, gangrene, and necrosis of subcutaneous tissues (including adjacent fascia). If the infection is not effectively controlled for a prolonged period, approximately 15% of patients will eventually require amputation, with a clinical mortality rate of approximately 10–30% [5].

During anti-infection treatment, conventional oral preparations, injections, and other routes of administration are commonly used for drug delivery because they can affect the entire body. However, systemic drug administration may result in low drug concentrations in localized lesions, and prolonged exposure to low drug concentrations can lead to bacterial drug resistance and other issues. Therefore, localized antimicrobial drug therapy is the preferred approach for treating SSTIs [6]. Traditional topical antimicrobial preparations (such as creams, ointments, and gels) are frequently employed to treat superficial skin diseases because of their ease of use and ability to provide a slow-release effect by forming a film on the skin surface. However, factors such as lipophilicity, molecular weight, molecular structure, and transdermal enhancers limit the transdermal efficiency of large molecules or hydrophilic drugs passing through the stratum corneum. Consequently, these agents cannot reach deeper infected areas of the skin [7].

In recent years, microneedle patches have been developed and recognized as promising systems for transdermal drug delivery because they are highly efficient, safe, and patient-compliant methods for topical drug delivery. They physically puncture the stratum corneum to form micropore channels, subtly bypassing most constraints imposed by the physicochemical properties of the drug and considerably improving the efficiency of transdermal drug delivery [8]. Dissolving-microneedle systems have also been developed and demonstrate potential in antimicrobial applications. However, despite the publication of several reviews addressing advancements in microneedle technology for drug delivery, a substantial gap persists in the comprehensive analysis of the advantages of dissolving microneedles in antimicrobial therapy and their current application status. Specifically, there is a notable absence of comparative analyses among the various types of antimicrobial dissolving microneedles, as well as thorough examinations of the challenges associated with their application, such as limited drug loading capacity, insufficient release control, biocompatibility issues, absence of standardized efficacy metrics, and difficulties in achieving sterile manufacturing.

**Scheme 1:**
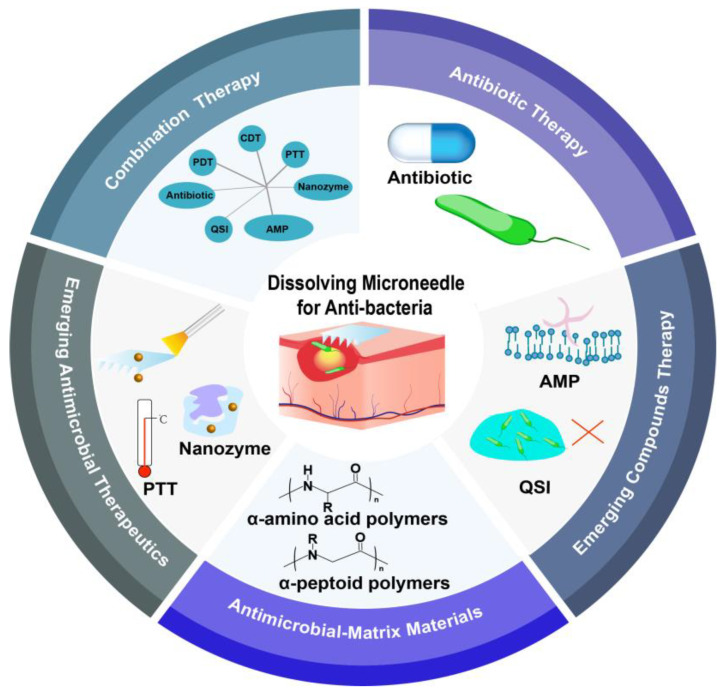
Schematic of various dissolving microneedles for bacterial-infection treatment.

Considering the above benefits and challenges, this review summarizes the advantages of diverse dissolving microneedles in antimicrobial therapy and discusses their current applications (Scheme 1). Specifically, it compares conventional antibiotic-based dissolving microneedles with emerging quorum sensing inhibitor (QSI) and antimicrobial peptide (AMP)-based dissolving microneedles. Additionally, we reviewed the frontier of antimicrobial dissolving microneedles that leverage antimicrobial-matrix materials, chemodynamic therapy (CDT), nanozyme-based therapy, photodynamic therapy (PDT), and photothermal therapy (PTT). We also highlight the potential of antimicrobial dissolving microneedles based on combination therapy to achieve enhanced efficacy. Finally, critically evaluates the clinical challenges, druggability, and prospective developmental pathways associated with antimicrobial dissolving microneedles. By comprehensively evaluating both conventional and emerging strategies using dissolving microneedles for antimicrobial purposes, this review aims to promote the advancement and clinical translation of microneedle technology while informing its future development.

## 2. Advantages and Challenges of Local Antimicrobial Therapies

### 2.1. Comparison of Systemic and Topical Antimicrobial-Therapy Strategies 

During anti-infection therapy, systemic administration typically involves the introduction of antimicrobial drugs into the bloodstream via oral or injectable routes to reach all parts of the body [9]. This approach offers the advantage of providing an effective treatment for systemic or multisite infections. However, it may also result in systemic side effects and contribute to the development of bacterial resistance [10]. For example, achieving a certain concentration of antimicrobial drugs in the body is necessary for effectiveness; however, this process can strain organs such as the liver and kidneys. Systemic administration leads to the widespread distribution of antimicrobial drugs throughout the body, exerting continuous selective pressure on bacterial populations and promoting the adaptation and reproduction of drug-resistant strains. Inappropriate dosages or regimens, as well as the excessive use of these drugs, increase bacterial resistance, thereby accelerating the emergence and spread of drug resistance [11].

Topical drugs are a class of medications that are applied to and work on the site of localized damage. They are characterized by their direct action on the target site of the disease and exert therapeutic effects [12]. Compared to systemic administration, topical drugs reduce systemic exposure, thereby avoiding liver first-pass and gastrointestinal side effects [13]. However, topical drugs can remain in the affected area for an extended period and maintain high local concentrations. This helps reduce excessive drug dosage and minimizes the development of drug resistance, thereby reducing the economic burden. Therefore, in clinical practice, antimicrobial drugs are often directly applied to infection sites using creams, ointments, gels, eye drops, ear drops, and nasal sprays [14]. Additionally, topical antimicrobial therapy can be combined with other adjunctive therapies, such as debridement and physiotherapy, to enhance therapeutic effectiveness and promote wound healing. 

### 2.2. Therapeutic Difficulties to Be Addressed in Topical Antimicrobial Therapy 

#### 2.2.1. Skin Barrier

**Figure 1:**
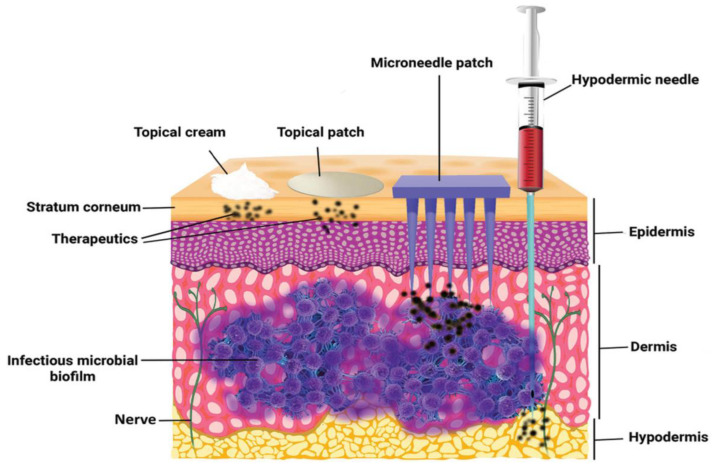
Skin structure and comparison of the skin-penetration depths of different drug-delivery systems. Reproduced with permission from [7] Copyrights 2020, WILEY.

The skin, the largest organ in the human body and the first natural barrier plays a crucial role in maintaining homeostasis and protecting the internal organs from the external environment [15,16]. The structure of the skin is divided into three layers (Figure 1): epidermis (100 μm), dermis (3–5 mm), and subcutaneous tissue. The epidermis, which is further subdivided into the stratum corneum, stratum pellucidum, stratum granulosum, and stratum basale, serves as the outermost layer of the skin. The main barrier to transdermal absorption of most drugs is the 10–15 μm-thick layer called the stratum corneum, composed of dead keratinocytes. Because of its barrier effect, only drugs with a relative molecular weight of less than 500 Da and suitable lipid solubility can easily pass through it. This limitation affects the efficiency of transdermal drug delivery, resulting in low bioavailability [17,18,19,20,21].

Infections deep in the skin, such as cellulitis involving the dermis and subcutaneous tissues, are typically more difficult to treat than epidermal infections. This is because conventional topical dosage forms, which are limited by the physical barrier of the skin, have a restricted ability to penetrate, resulting in insufficient drug concentrations at the site of infection for the complete elimination of infectious pathogens (Figure 1) [7]. Therefore, high doses of antimicrobial drugs are typically administered to treat deep-seated infections and achieve the desired therapeutic effects [21,22].

#### 2.2.2. Bacterial Resistance

**Figure 2:**
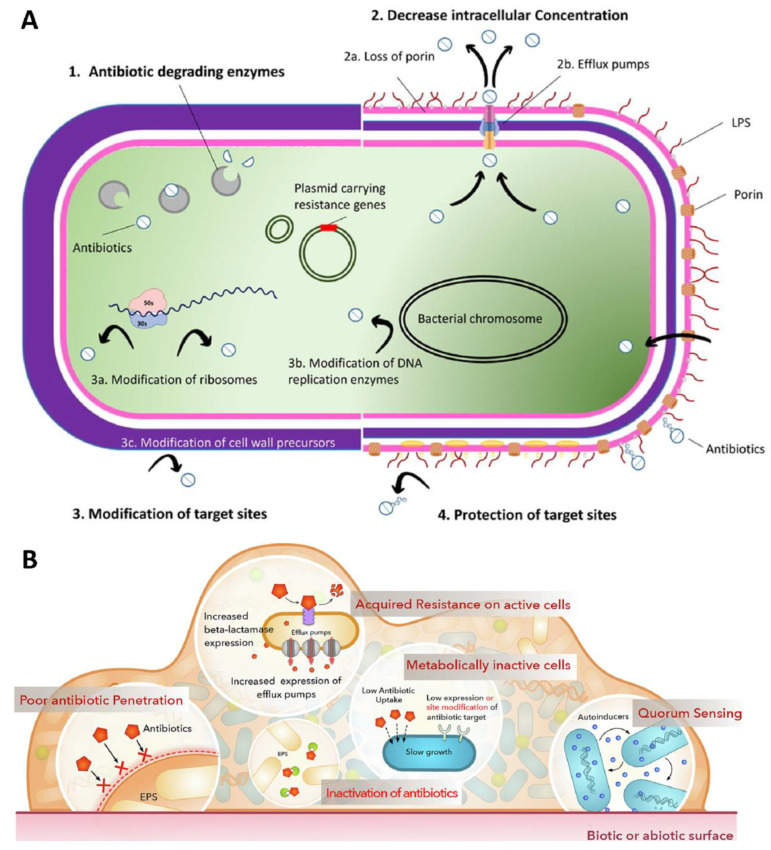
Mechanism of bacterial and biofilm resistance. (**A**) Molecular mechanisms of antibiotic resistance [25]. Reproduced with permission from [25] Copyrights 2022, Advanced Drug Delivery Reviews. (**B**) Mechanisms of antimicrobial resistance of biofilms [26]. Reproduced with permission from [26] Copyrights 2023, Journal of Controlled Release.

The increase in bacterial resistance is a growing health problem worldwide, and the World Health Organization has ranked antibiotic resistance as one of the top 10 global public health threats [23]. The emergence of drug resistance is a multifactorial process involving multiple biochemical pathways and mechanisms (Figure 2A). For example, bacteria can acquire various antibiotic-resistance genes through horizontal gene transfer. Additionally, bacterial cell-membrane permeability is reduced, bacteria produce degradative enzymes that break down antibiotics, antibiotics are inactivated by the increased production of antibiotic-modifying enzymes, and antibiotics are rendered unable to bind because of bacterial modification of the target site through genetic modification. Moreover, exocytosis pumps are overexpressed, leading to rapid drug efflux. Other factors include the expression of regulatory small RNAs and methylation of 16 S and 23 S rRNAs by methyltransferases, thereby altering antibiotic binding sites and leading to changes in the bacterial metabolic state, formation of biofilms, and emergence of persister cells [24].

Additionally, biofilm formation is an important factor that cannot be ignored because of the increase in bacterial-drug resistance (Figure 2B). Over 70% of human bacterial infections, both recalcitrant and chronic, are associated with biofilm formation. Biofilms are complex, three-dimensional community structures formed by bacteria on solid surfaces and are surrounded by extracellular polymeric substances (EPS). The protective EPS of biofilms can effectively act as physical and metabolic barriers that render bacteria highly resistant to antibiotics through various mechanisms, such as limiting antibiotic penetration, inducing antibiotic inactivation via enzymatic and chelation reactions, enhancing community sense, and increasing the activity of bacterial drug efflux pumps [27].

Bacteria encased in biofilms have been reported to be 10–1000 times more resistant to antibiotics than planktonic bacteria, and completely eradicating bacteria in biofilms using conventional antibiotic therapies is challenging. In addition, the presence of biofilms increases the recurrence rate of infection and complicates treatment. The challenge in treating biofilm-associated infections lies in overcoming the natural barrier provided by the biofilm while designing therapeutic regimens to target bacterial populations inside the biofilm that may be in different metabolic states [28]. New therapeutic approaches are being explored, including the use of nanotechnology to improve drug penetration and targeting, develop enzymes capable of breaking down extracellular polymers, utilize alternative therapies, such as phages and AMPs, and enhance the host’s own immune response [29]. Furthermore, the early diagnosis and real-time monitoring of biofilm formation are crucial for improving therapeutic efficacy [30]. These strategies should integrate the complexity of biofilms and bacterial adaptations to develop more effective treatments.

## 3. Advantages and Classification of Microneedle Systems

In 1976, a new technology called microneedles was proposed to overcome the limitations of conventional transdermal drug delivery. Since the 1990s, with the development of modern fine processing technology, many types of microneedles have been successfully prepared [19]. Microneedles combine the advantages of injectable and transdermal drug-delivery systems. They generally consist of multiple micrometer-sized needle tips attached to a substrate base in an array. Their body length ranges from approximately 25 μm to 2000 μm, and their width is approximately 10–50 μm [31,32,33]. The length of their micrometer-sized bodies allows them to break through the stratum corneum barrier and form a micrometer-sized drug-delivery channel between the skin surface and dermis, delivering high-molecular-weight and highly hydrophilic drugs to the skin dermis. Drugs can be absorbed through skin microcirculation for systemic effects. Because the tip of microneedles is not long enough to touch nerve fibers in the epidermis or dermis, their use for drug delivery does not cause significant pain. 

**Figure 3:**
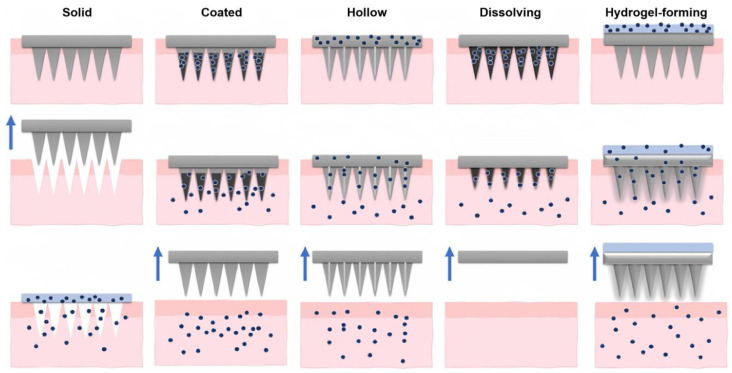
Five types of microneedles (solid, coated, hollow, dissolving, and hydrogel) developed to date. Reproduced with permission from [35] Copyrights 2022, Materials Today Chemistry.

Microneedles are widely used as an attractive delivery system for the convenient, noninvasive, and painless delivery of diverse therapeutic agents of various sizes, such as small-molecule compounds, peptides, proteins, DNA, and vaccines, to the dermis and into the body circulation [21,34]. Different types of microneedles have been developed, including solid, hollow, coated, hydrogel, and dissolving microneedles (Table 1 and Figure 3) [20].

### 3.1. Solid Microneedles

Solid microneedles are primarily used for skin pretreatment by inserting and removing them to create micrometer-sized pores on the skin surface. The drug slowly diffuses into the body through these microneedles after application to the skin, thereby enhancing its permeability. Factors such as microneedle insertion force, sharpness, and density play important roles in determining the performance of solid microneedles.

Solid microneedles are commonly prepared from silicon; non-degradable materials such as lithographic epoxy, polycarbonate, and polymethylmethacrylate; degradable polymers such as polylactic acid and polyglycolic acid; metals including stainless steel, titanium, and nickel; and ceramics. The common preparation methods include laser cutting and chemical etching. The manufacturing process focuses on selecting the appropriate microneedle material and geometry to provide sufficient mechanical strength while increasing the sharpness of the tip to reduce the force required for insertion into the skin tissue [18].

Solid microneedles do not carry drugs themselves but only create micro-orifices; thus, they have certain limitations. First, the micro-orifices left behind after removing the microneedle may allow toxic substances, such as pathogenic microorganisms, to invade and increase the risk of infection. Second, the contraction and blockage of microchannels can hinder molecular delivery. Third, precisely controlling the dosage for drug delivery is challenging, and the procedure itself is complex. Finally, if needle breakage occurs, retaining the needle in the patient’s body poses a safety hazard [36].

### 3.2. Hollow Microneedles

Hollow microneedles are miniaturized versions of conventional hypodermic needles with a cavity inside each needle body on the baseplate, allowing drug solutions to be injected into the skin through diffusion, pressure, or electrically driven flow. Hollow microneedles are primarily made from materials such as silicon, metal, glass, polymers, and ceramics using advanced technologies such as microelectromechanical systems, ion etching, wet chemical etching, deep X-ray lithography, integrated photolithography molding, and three-dimensional (3D) printing.

Compared to solid microneedles, hollow microneedles utilize pressure-driven drug delivery, which offers the advantages of rapid drug administration, continuous infusion, and an adjustable release rate [37], thereby facilitating precise drug delivery. However, the efficiency of drug delivery using hollow microneedles may be affected by channel obstruction. Tissue blocks the needle tip during skin insertion [38]. In contrast, the dense dermal tissue surrounding the microneedle tip creates resistance to flow owing to compression [39]. Additionally, commonly used materials, such as silicon and glass, are brittle substances that can easily cause needle tips to break and remain in the body if excessive force is applied after skin penetration, posing certain safety risks. Thus, the preparation of hollow microneedles is challenging because of their brittle structure and materials [40].

Recently, Liu et al. [41] designed a biomimetic microneedle patch known as HepMi-PCL, which utilizes 3D printing technology to fabricate hollow microneedles. Each needle has an equally sized porous shell and a cavity filled with heparin-based functional hydrogel (Hep). The water-absorbing ability of Hep allows the microneedle to perform integrated diagnostic and therapeutic functions by monitoring changes in exudate at the site of infection. In vivo pharmacodynamic studies have shown that the degree of wound healing on the dorsal surface of rats in the HepMi-PCL group has exceeded 90% following 14 days of treatment.

### 3.3. Coated Microneedles

Coated microneedles, also known as coated microneedle systems, are a type of microneedle system in which a drug solution is applied to the surfaces of solid microneedles. This system is particularly suitable for the rapid delivery of high-molecular-weight molecules (e.g., vaccines, proteins, peptides, and DNA) to the skin [42]. Various strategies have been developed for coating and drying drug molecules. (1) Spraying method: This method achieves coating by uniformly spraying drug droplets onto the entire surface of a microneedle patch. Although this allows for the mass production of coated microneedles, a significant amount of the drug is lost during preparation and application. (2) Inkjet printing: This novel preparation technique ensures uniform drug loading with minimal variation between different samples. However, this method is time-consuming and requires further research and improvement for large-scale applications. (3) Dip-coating method: This method utilizes a dip coater with micro-positioning capabilities to coat the drug solution onto microneedles. Although this process is commonly used in manufacturing, challenges remain regarding precise dosage control, content uniformity, and large-scale production [43,44,45].

Adding viscosity enhancers, surfactants, or other excipients to the drug solution used to coat the microneedles is typically necessary to adhere the solution to the surface of the microneedles. However, this not only reduces the drug-carrying capacity of the microneedle patch and affects the sharpness of the microneedle tip but may also pose a safety hazard; therefore, this method is rarely used [46].

### 3.4. Hydrogel Microneedles

Hydrogel microneedles are typically prepared from hydrogels with a hydrophilic cross-linked structure or a super-swellable polymer matrix that swells subcutaneously without dissolving [38,47]. Commonly used materials include poly(methyl vinyl ether-alt-maleic acid) polymers, poly(methyl vinyl ether*-co-*maleic anhydride), hyaluronic acid methacrylate, and polyvinyl alcohol.

Two primary modes of transdermal drug delivery exist for hydrogel microneedles. The first method involves loading the drug into a hydrogel microneedle, which swells upon insertion into the skin due to the absorption of interstitial fluid. This allows the therapeutic drug to diffuse through the hydrogel polymerization network and be absorbed by dermal microcirculation, achieving systemic absorption [48]. In the second approach, the drug is attached to a reservoir in the basal layer, and the microneedle itself does not contain any drugs. After insertion into the skin, rapid interstitial fluid absorption creates a continuous channel between the dermal microcirculation and attached patch-type drug reservoir, enabling drug diffusion from the reservoir layer into the dermal microcirculation. This drug-delivery system no longer limits the delivered dose of drugs and biomolecules based on needle capacity alone [49].

Furthermore, interstitial fluid contains biomarkers similar to those found in the plasma and is considered a significant source of biomarkers. The interstitial fluid absorbed by hydrogel microneedles can be retrieved from the microneedles by centrifugation or heating for subsequent biomarker analyses [50]. Therefore, hydrogel microneedles can also be utilized in minimally invasive diagnostics, which is unfeasible with other types of microneedles.

However, hydrogel microneedles face challenges in practical applications. Drug release from hydrogels involves both burst and steady-state release processes. If the burst effect during hydrogel administration is not controlled, drug concentrations in the serum may exceed the toxicity limit, causing local tissue damage [51]. Second, if the polymer used to prepare the hydrogel is insufficiently crosslinked, it may dissolve upon contact with the interstitial fluid. If a dissolved polymer is cytotoxic, toxicity may accumulate in vivo [52,53].

### 3.5. Dissolving Microneedles

Dissolving microneedles are drug-delivery systems that use biodegradable polymer-matrix materials. Drugs are loaded into their microneedle tips, and when the microneedles puncture the skin, they are released as the polymer matrix dissolves [54,55,56]. Compared to solid microneedles, hollow microneedles, coated microneedles, and hydrogel microneedles, dissolving microneedles have the following advantages. (1) They can quickly dissolve in the interstitial fluid of the skin, thereby shortening the treatment-process time. (2) They are easy to administer and increase patient compliance. (3) They avoid the risk of hazardous sharp biowaste residue in the skin, reduce damage to skin tissues, and provide a higher level of safety. (4) The risk of blood-borne infections and cross-contamination is avoided. (5) The precision of the drug-delivery dosage can be ensured by separating the tip of the microneedle from the basal layer. (6) The regulation of drug-release time can be achieved by controlling the dissolution rate of the microneedle material. (7) The preparation process is simple and inexpensive.

Currently, polymeric materials used for the preparation of dissolving microneedles are mainly classified into natural polymeric materials (including sodium hyaluronate, chondroitin sulfate, carboxymethyl cellulose, sodium alginate, dextran, and maltose) and synthetic polymeric materials (such as polyvinylpyrrolidone, poly(vinylpyrrolidone*-co-*methacrylic acid), and polymers including polylactic acid, polyglycolic acid, and poly(vinylpyrrolidone*-co-*methacrylic acid) [57]. Depending on the nature of the polymer material, several techniques are available for preparing dissolving microneedles. The main preparation methods include solvent casting, stretch lithography, droplet-blowing laser processing, thermocompression micro-injection molding, and ultrasonic welding. Solvent casting is the most commonly used preparation method. In this method, the drug and excipient components are first dissolved in a suitable solvent, filled into the microneedle mold cavity, and finally molded by drying [58]. The advantage of this method is that it does not require harsh processing conditions, such as high temperatures, making it easy to prepare [59].

Dissolving microneedles have broad application prospects in the field of drug delivery, and 15 products related to dissolving microneedles entered clinical trials since 2007 (Table 2) [36]. Dissolving microneedles can easily achieve multiple therapeutic functions by loading different components or combining them for various therapeutic strategies [36,60,61]. Currently, dissolving microneedles are widely used in cosmetology, vaccines, chronic diseases, and tumor treatment. For example, in cosmetology, dissolving microneedles are used to deliver anti-aging ingredients and skin-repairing substances to promote skin regeneration and firmness. Park et al. [62] prepared cosmetic microneedle patches using sodium hyaluronate as a microneedle matrix material and branched starch to adjust the mechanical strength and solubility of the microneedles by loading cosmetic ingredients such as niacinamide and ascorbic acid into them. The experimental results showed that the permeability of niacinamide in the skin and the antioxidant activity of ascorbic acid were significantly enhanced after crossing the skin. Dissolving microneedles can deliver vaccines intradermally, improving the effectiveness of the immune response and reducing the discomfort and side effects associated with conventional injections. Donadei et al. [63] developed a dissolving microneedle containing a trivalently inactivated poliovirus vaccine with trehalose and polyvinyl alcohol, which induced neutralizing antibody responses comparable to those of intramuscular injections of higher doses of poliovirus vaccine types 1 and 3. Dissolving microneedles can be used for long-term drug delivery to improve drug adherence and therapeutic efficacy in the treatment of chronic diseases. Jana et al. [64] prepared insulin-loaded dissolving microneedles using modified sodium carboxymethylcellulose and gelatin, which possessed excellent mechanical strength and a subcutaneous diffusion range of more than 750–800 µm. The in vivo experimental results showed that the same dose of insulin-loaded microneedles was comparable to that of subcutaneous insulin and that the release process was more sustained and beneficial for stabilizing blood glucose levels.

## 4. Advancements in Dissolving Microneedles Incorporating Conventional Antibiotics

Since the discovery of penicillin in 1929, numerous types of antibiotics have been developed to reveal the interactions between drug targets and modifying drug molecules. These include fluoroquinolones (such as ciprofloxacin and levofloxacin), rifamycins (such as rifampicin and rifapentine), β-lactams (including penicillin G, benzoxirin, cefradine, ceftriaxone, cefepime, and imipenem), glycopeptides (such as vancomycin and teicoplanin), polypeptides (such as daptomycin and polymyxin B), aminoglycosides (including gentamicin, tobramycin, streptomycin, and kanamycin), tetracyclines (such as tetracycline and doxycycline), macrolides (such as roxithromycin and azithromycin), and lincomycins (including chloramphenicol and clindamycin) [65,66]. Soluble microneedles have been used to deliver several antibiotics, with good antimicrobial and therapeutic results.

Xu et al. [67] developed a dissolving microneedle that contained chloramphenicol- and gelatinase-sensitive gelatin nanoparticles. When the microneedle was applied to the site of infection, it dissolved and uniformly released chloramphenicol gelatin nanoparticles. These nanoparticles decomposed in the presence of gelatinase produced by live bacteria, releasing chloramphenicol into the active region of the biofilm and effectively eliminating it. Compared with direct administration, this approach minimizes the off-target toxicity of chloramphenicol nanoparticles, thereby facilitating wound healing. This therapeutic strategy has great potential for improving the delivery of various antimicrobial agents to biofilm sites.

Abdelghany et al. [68] prepared dissolving microneedles loaded with ciprofloxacin and investigated their potential for treating SSTIs caused by *S. aureus*. In an agarose model of *S. aureus* infection, the microneedle formulation exhibited significantly enhanced antimicrobial activity against *S. aureus* compared to the ciprofloxacin gel (*p* < 0.0001). Permana et al. [69] developed a dissolving microneedle designed to incorporate doxycycline (DOX)-loaded nanoparticles that are sensitive to bacteria. These nanoparticles are composed of poly(lactic*-co-*glycolic acid) and polycaprolactone (PCL) and are decorated with chitosan to enhance their antimicrobial and antibiofilm activity. In vitro biofilm-activity assays demonstrated a significant reduction in bacterial bioburden, with a 99.99% decrease after 48 h. Moreover, the dissolving microneedle is capable of penetrating bacterial biofilms in two ex vivo biofilm models in full-thickness porcine skin and delivering DOX-loaded nanoparticles to deep infection sites. This process enhances the dermatokinetic profiles and antibiofilm activities of DOX.

Cellulitis is a chronic wound infection located in the dermal/subcutaneous layer of the skin, caused by the immune system (cytokines and neutrophils) reacting to bacteria that penetrate the epidermis. Bacterial strains at the site of infection typically secrete lipolytic esterases, which cause the biocatalytic hydrolysis of PCL. Mudjahid et al. [70] used PCL to construct chloramphenicol microparticles and further incorporated the microspheres into microneedles for the treatment of cellulitis. In an ex vivo model of infection on rat skin, treatment with MPs-CHL DMNs for 24 h resulted in a reduction of bacterial bioburdens by 99.99%. The microneedle group showed significant therapeutic advantages over the ointment group with the same drug composition.

## 5. Dissolving Microneedles Based on Other Promising Antimicrobial Drugs

### 5.1. Antimicrobial Dissolving Microneedles Based on QSIs

Quorum sensing (QS) is a specific signaling mechanism between microbial cells in which bacteria utilize a quorum-sensing molecular communication system to regulate cellular metabolic activities, promote biofilm formation, and enhance virulence [24]. As promising anti-biofilm drugs, QSIs can exert their inhibitory effects on community sensing by suppressing the synthesis of signaling molecules, directly degrading signaling molecules, inhibiting the binding of signaling molecules to receptors, and/or blocking signal transduction cascades [71,72,73,74,75]. Based on their backbone, QSIs can be categorized into furanone derivatives, benzothiazole derivatives, pyranone derivatives, quinolines, pyrrole derivatives, indole derivatives, pyridine derivatives, ester derivatives, pyrimidines, thiazole and thiadiazole derivatives, peptides and others [76,77].

Chen et al. [78] designed a dissolving microneedle containing the flavonoid QSI luteolin to treat bacterial biofilm-associated wound infections. The matrix material of the microneedle was sodium hyaluronate, and the tip of the needle contained both the QSIs luteolin and a nanomotor composed of the photosensitizer indocyanine green and nitric oxide donor L-arginine. When applied to an infected wound, the microneedle rapidly dissolves due to sodium hyaluronate, releasing the QSIs luteolin, which damages bacteria and exerts anti-biofilm activity by inhibiting biofilm formation. Under near-infrared irradiation, both photothermal and nitric oxide drive the nano-motor to move more effectively within an already disturbed biofilm, further enhancing multiple anti-bioinfection effects, including PTT, PDT, and nitric oxide. In vivo experiments demonstrated that the microneedles promoted traumatic granulation-tissue formation and collagen-fiber deposition, thereby accelerating wound healing.

Disruption of the bacterial QS pathway is considered an effective approach to address bacterial antibiotic resistance because of the critical role of QS in the formation of bacterial virulence. Alasiri et al. [71] screened a variety of naturally occurring small-molecule compounds to disrupt the QS pathway in *Pseudomonas aeruginosa* (*P. aeruginosa*) and performed a series of in vitro correlation assays on the drug candidates. Bakuchiol (Bak) inhibits the formation of *P. aeruginosa* biofilm and virulence factors. In a LuxR-type receptor assay, Bak selectively inhibits LasR in *P. aeruginosa* in a dose-dependent manner. This may be because Bak destabilizes LasR and disrupts its functional quaternary structure upon binding. The determination of the inhibition produced by virulence factors showed that Bak inhibited pusillanimals and rhamnolipids by 71.5% and 66.9%, respectively, compared to the reference inhibitor (percentage inhibition of 52.7% and 57.7%, respectively). This indicates significant inhibitory effects, making it a promising and potent anti-biofilm agent against *P. aeruginosa*.

### 5.2. Antimicrobial Dissolving Microneedles Based on AMPs

AMPs, also known as host defense peptides (HDPs), are a class of small-molecule peptides consisting of 12–50 amino acid residues. They are structurally composed of mainly positively charged amino acid residues, such as lysine, arginine, and histidine, as well as hydrophobic amino acid residues (approximately 50%) [79,80,81]. AMPs are widely present in various organisms and serve not only as important effectors of the innate immune system but also as the first line of defense against pathogenic infections [82]. According to the AMP database, more than 3100 natural AMPs have been identified to date.

Unlike conventional antibiotics, which act on intracellular structures, AMPs mainly exert their antimicrobial effects by disrupting the bacterial–cell membrane [79]. This mechanism does not involve site-specific binding or interference with bacterial metabolism and is, therefore, less likely to result in drug resistance. The antibacterial mechanism of AMPs consists of several key elements. First, positively charged amino acid residues in the peptide interact electrostatically with the anionic bacterial membrane, stabilizing the binding of the peptide to the cell membrane. Subsequently, its hydrophobic structural domains are inserted into the lipid bilayer, disrupting the bacterial–cell membrane and forming pores that lead to alterations in the bacterial–cell membrane potential, changes in membrane permeability, and leakage of the contents, ultimately resulting in bacterial cell death [83]. Because the outer layers of mammalian cells are dominated by lipids with zero net charge (e.g., phosphatidylcholine and cholesterol exhibit electroneutral properties), AMPs are more inclined to bind to bacteria [84,85].

Because of their broad-spectrum antibacterial properties, low biotoxicity, and unique mechanism of membrane disruption, AMPs are considered an effective solution to the problem of resistance to conventional antibiotics and are expected to serve as an alternative [86,87]. Wang et al. [88] utilized the cationic nature of chitosan and anionic properties of gum arabic to prepare nanoparticles for encapsulating AMPs. They further developed dissolving microneedles by incorporating the encapsulated nanoparticles into recombinant human collagen III to construct a drug-delivery system for bacterial clearance and wound healing. The system facilitated transdermal drug delivery through microneedles and delivered drug-carrying nanoparticles into the subcutaneous tissue. Under microenvironmental conditions characterized by acidic pH during bacterial infection, electrostatic interactions between chitosan and gum arabic were weakened, altering the structure of the nanoparticle networks and contributing to the slow release of AMPs. In vivo experiments conducted on *S. aureus*-infected mice demonstrated that compared with other groups receiving different forms of drug administration, mice treated with drug-loaded microneedles exhibited increased vascularization in traumatic tissues and optimal wound healing.

Inspired by the classical amphiphilic helical structure of LL−37, Su et al. [89] designed a short-chain AMP, W379, with alkaline-charged amino acids at its top and hydrophobic structural domains at its bottom. This peptide was then incorporated into electrostatically spun nanofibers and dissolving microneedles to create Janus-type dressings for eradicating bacterial biofilms from chronic wounds. The dressing utilized the physical penetration of microneedles to deliver the AMP into the inner biofilm and disrupt its structure. However, the nanofiber membrane in the dressing continuously released the AMP to target and destroy the outer biofilms, exerting a bidirectional antimicrobial effect. In vivo experiments demonstrated that no bacteria survived within the biofilm after treatment with Janus-type dressings, indicating a significant wound-healing effect.

However, multiple doses are typically required for complete biofilm removal during wound infection treatment. Based on the exploration of the AMP W379, Su et al. [90] designed a microneedle patch with a photothermally triggered release of the AMP. The surface of the microneedle was coated with a layer of 1-tetradecanol with phase-change properties, and its interior was loaded with an AMP and a photothermal agent. When the microneedle physically penetrates the biofilm, near-infrared light irradiation can stimulate the photothermal conversion effect of the agent, resulting in the melting of the phase-change material and triggering the release of the AMP. By controlling the duration of light exposure, precise regulation of AMP release can be achieved, leading to enhanced antimicrobial and antibiofilm effects. In vivo experimental studies have shown that after four rounds of light exposure within 48 h, bacteria were completely killed using drug-loaded and coated microneedles, which promoted wound healing and reduced the discomfort caused by frequent drug administration.

## 6. Dissolving Microneedles Constructed from Antimicrobial-Matrix Materials

Currently, the application of dissolving microneedles in the field of antimicrobials is limited by several factors: (1) Only a few types of excipients are suitable for preparing antimicrobial microneedles; (2) excessive excipients in microneedles typically occupy the space for drug loading, which leads to difficulty in achieving greater efficacy with concentration-dependent antimicrobials; (3) high antibiotic loading may induce the formation of poorly water-soluble drug crystals and accumulate stresses at the tip of microneedles, increasing the risk of needle breakage; (4) conventional small-molecule antibiotics have poor retention ability at infection sites, making the long-term inhibition of local skin infections difficult; (5) mechanisms through which drug-resistant bacteria reduce intracellular antibiotic concentrations may further increase the difficulty in treating localized infections [91,92,93,94].

Recently, dissolving microneedles prepared from materials with antimicrobial properties have been reported. This strategy is based on replacing conventional dissolving microneedle matrix materials with inherently antimicrobial polymers. This strategy offers the following advantages: (1) When preparing microneedles with antimicrobial-matrix materials, the risk of bacterial infections due to the creation of microporous channels in the microneedles can be further avoided owing to the antimicrobial activity of the matrix materials; (2) when preparing dissolvable antimicrobial microneedles using antimicrobial-matrix materials, the amount of antimicrobial drugs used can be reduced by utilizing the antimicrobial activity of the matrix materials to avoid impacting the mechanical strength and stability caused by excessive drug loading.

### 6.1. Recent Advances in Naturally Derived Antimicrobial Materials

Chitosan is one of the most extensively studied antimicrobial cationic polymers and exhibits broad-spectrum bactericidal activity against both Gram-negative and Gram-positive bacteria. It possesses several advantages, such as biodegradability, non-toxicity, and high biocompatibility. Because of its positive charge, chitosan can interact with anionic groups on bacterial cell walls or membranes through numerous protonated amino groups (–NH^3+^), thereby altering membrane permeability and causing membrane rupture. In addition, chitosan can penetrate bacterial cells and disrupt their normal physiological activities, ultimately leading to bacterial death. Chi et al. [95] incorporated a vascular endothelial growth factor (VEGF) into the micropores of chitosan microneedle arrays using a temperature-sensitive hydrogel called poly(N-isopropylacrylamide). This hydrogel controlled the release of VEGF in response to an increase in the body-surface temperature resulting from inflammation at the wound site. The findings demonstrated that these microneedles effectively delivered high doses of VEGF to the wound site, inhibited inflammation, and promoted wound healing, neovascularization, and tissue regeneration. However, limited research has been conducted on chitosan microneedles owing to their poor water solubility. Recent studies have addressed this issue by employing various chemical modifications, such as graft co-polymerization, quaternization, acylation, and alkylation [96]. These modifications improve the aqueous solubility of chitosan by introducing hydrophilic groups and disrupting intramolecular or intermolecular hydrogen bonds. It is expected that these methods will enhance the interaction between chitosan derivatives and bacterial cells as well as regulate their release behavior. Consequently, these water-soluble chitosan derivatives are anticipated to be a good alternative for excipients in the preparation of microneedles based on chitosan.

ε-Poly-*L*-lysine (EPL) is a natural cationic antimicrobial agent produced by Streptomyces albicans, consisting of 25–35 lysine residues. It exhibits antimicrobial activity against a wide range of microorganisms, including Gram-positive and Gram-negative bacteria [97]. Owing to its broad-spectrum antimicrobial activity, biodegradability, and biocompatibility, it is currently approved by the U.S. Food and Drug Administration as a food-grade preservative [98]. EPL consists of the natural amino acid L-lysine but is linked by an isopeptide bond between the ε-amino and α-carboxylic groups. This gives it structural characteristics that are different from those of natural α-peptides. With favorable polymer structural properties (25–35 degrees of polymerization, 3.2–4.5 kDa), EPL can potentially be used in the development of dissolving microneedles as both antimicrobial agents and matrix materials.

**Figure 4:**
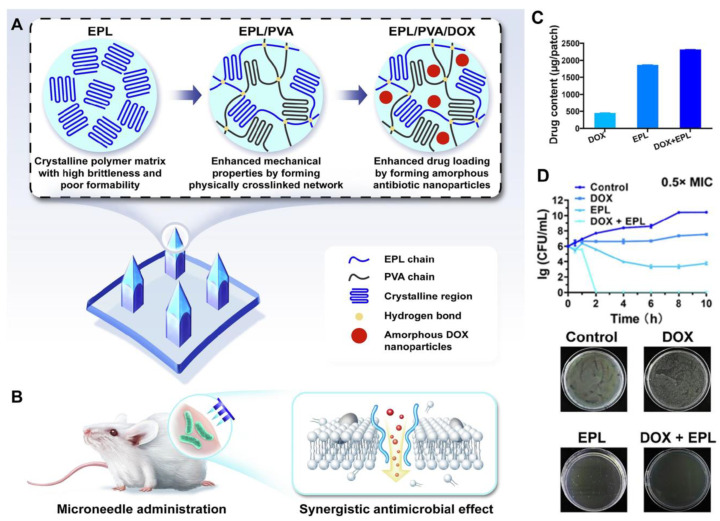
Dissolving microneedles constructed from antimicrobial-matrix materials. (**A**) Schematic illustration depicted the formation mechanism of physically crosslinked networks and amorphous antibiotics in the DOX-loaded EPL microneedles. (**B**) Synergistic antibacterial mechanism of DOX and EPL. (**C**) Drug loading of DOX and EPL in the microneedles. (**D**) Time–Kill curve of DOX and EPL combination at 0.5 × MIC. Reproduced with permission from [100] Copyrights 2024, International Journal of Biological Macromolecules.

Recently, Jiang et al. [99] investigated the feasibility of using EPL to construct dissolving microneedles by exploiting its broad-spectrum antimicrobial activity and excellent mechanical properties. Microneedles made solely from EPL have a high rate of needle breakage. However, by optimizing the addition of polyvinyl alcohol (PVA), microneedles can be produced with enhanced tip formability and high EPL loading capacity. Subsequently, Luo et al. [100] constructed a dissolving microneedle made of EPL and antibiotic DOX to treat bacterial skin infections (Figure 4). The physical cross-linking network formed by EPL and PVA in the tip provided spatially limiting domains effect and provided multiple intermolecular interactions, which not only improved the formability and mechanical properties of the microneedle tip but also inhibited the crystallization of the insoluble DOX as 7.2 nm amorphous nanoparticles. The obtained DOX-loaded EPL microneedles exhibited good formability, and the total antimicrobial loading in each patch was as high as 2319.1 μg, significantly higher than that of conventional antibiotic microneedles. When administered via the microneedle, positively charged EPL can penetrate the infected tissue and alter bacterial-cell membrane permeability, thereby promoting the intracellular accumulation of antibiotics and exerting a combined antimicrobial effect. The in vivo results demonstrated that deep infections induced by *P. aeruginosa* treated with the DOX-loaded EPL microneedles resulted in 99.91% bacterial clearance without significant toxic side effects.

### 6.2. Recent Advances in Synthetic Antimicrobial Materials

In addition to natural sources of antimicrobial polymers, synthetic polymers are also important sources of antimicrobial materials. Polyionic liquids (PILs) are a class of polymers with high physical and chemical stability and inherent antimicrobial properties owing to the presence of cationic groups, such as imidazole, pyrrolopyridine, and quaternary ammonium. They can be used as both carrier materials and antimicrobial drugs. Zhang et al. [101] developed microneedles based on salicylic acid, active pharmaceutical ingredients, and PILs that could be used to treat acne infections. In this study, PIL microneedles were prepared via photocrosslinking curing, followed by immersion in a saturated sodium salicylate solution for 3 days, resulting in the binding of salicylate anions to PIL cations through electrostatic interactions. The resulting drug-loaded microneedle patches exhibited high mechanical strength and antimicrobial properties against *Escherichia coli (E. coli)*, *S. aureus*, and *Streptococcus acnes*.

Gantrez^®^ AN is a biodegradable anhydride copolymer that contains alternating maleic anhydride groups and methyl vinyl ether groups, exhibiting good antimicrobial properties. Boehm et al. [102] fabricated microneedles using the anhydride copolymer Gantrez^®^AN 169 BF. In vitro antimicrobial experiments demonstrated the effectiveness of these microneedles against a wide range of microorganisms, including *Bacillus subtilis*, *Enterococcus faecalis*, *E. coli*, and *S. aureus*.

**Figure 5:**
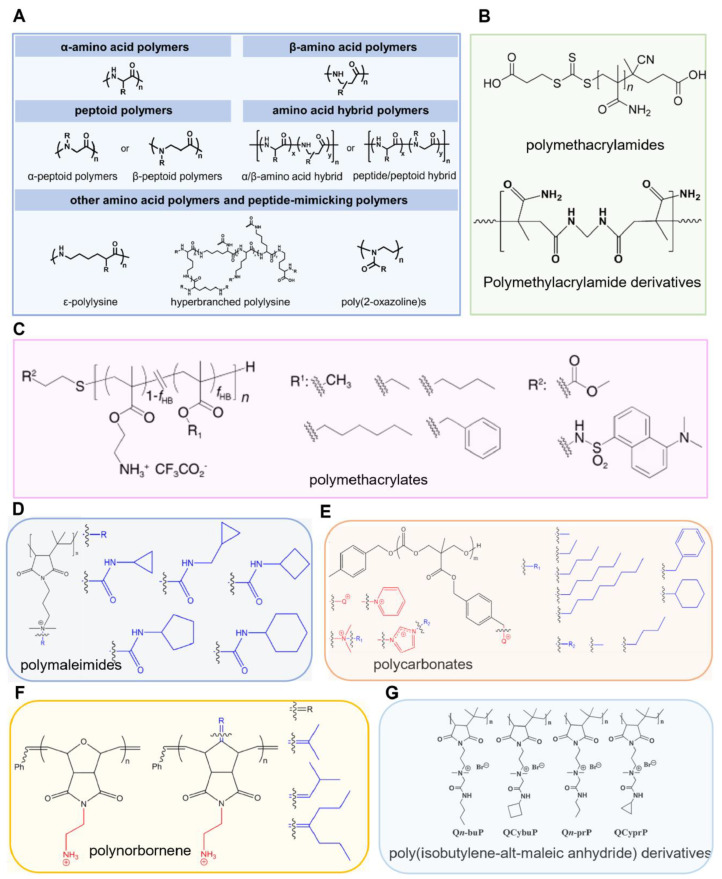
Structures of various AMP-mimicking antimicrobial polymers. (**A**) Structures of HDP-mimicking amino acid polymers. Reproduced with permission from [120] Copyrights 2023, Progress in Polymer Science. (**B**) Structures of polymethacrylamide derivatives. Reproduced with permission from [109] Copyrights 2014, Biomaterials. (**C**) Structures of polymethacrylate derivatives. Reproduced with permission [110] from Copyrights 2009, Chemistry-A European Journal. (**D**) Structures of polymaleimides derivatives. (**E**) Structures of polycarbonate derivatives. (**F**) Structures of peptidopolysaccharide derivatives. (**D**–**F**) Reproduced with permission from [113] Copyrights 2018, Biomacromolecules. (**G**) Structures of poly(isobutylene-*alt*-maleic anhydride) derivatives. Reproduced with permission from [118] Copyrights 2013, Chemical Communications.

In recent years, polymers with structural features mimicking AMPs have also garnered significant attention (Figure 5), which can be categorized as poly(α-amino acid)s [103,104,105], poly(β-amino acid)s [106,107], poly(2-oxazoline) [108], polymethacrylamide [109], polymethacrylate [110,111,112], polymaleimides [113], polycarbonates [114,115], peptidopolysaccharide [104], polynorbornenes [116,117], and poly(isobutylene-alt-maleic anhydride) derivatives [118,119]. These antimicrobial-mimicking peptides exhibit high antimicrobial activity and molecular weights and possess the properties of both an antimicrobial drug and a polymer excipient. They can be utilized as biomaterials or medicinal excipients in the construction of antimicrobial microneedles [120,121].

## 7. Dissolving Microneedles Based on Emerging Antimicrobial Therapeutics

### 7.1. Antimicrobial Dissolving Microneedles Based on CDT

CDT is an emerging therapeutic approach, and its core principle is to catalyze the generation of toxic hydroxyl radicals (–OH) or other cytotoxic substances from hydrogen peroxide (H_2_O_2_) in the environment through a Fenton or Fenton-like reaction, thereby achieving a destructive effect on the target cells [122,123,124]. Fenton reactions typically involve iron ions, whereas Fenton-like reactions can utilize other metallic elements such as copper, manganese, cobalt, molybdenum, platinum, tungsten, nickel, silver, ruthenium, vanadium, gold, zinc, and other elements [125].

Notably, CDT is typically associated with the reduction of metal and hydrogen-peroxide levels. However, the different wound environments caused by bacterial infections may not provide optimal conditions for this response, thereby limiting the therapeutic efficacy of single-metal ion-mediated CDT. To overcome this limitation, researchers are developing redox pairs of metal ions or designing self-supplied hydrogen peroxide systems to enhance the effectiveness of CDT. In addition to utilizing CDT alone, it can be combined with other therapies such as PTT, starvation therapy, PDT, and acoustic dynamics to achieve a synergistic antimicrobial system that meets various therapeutic needs [124].

**Figure 6:**
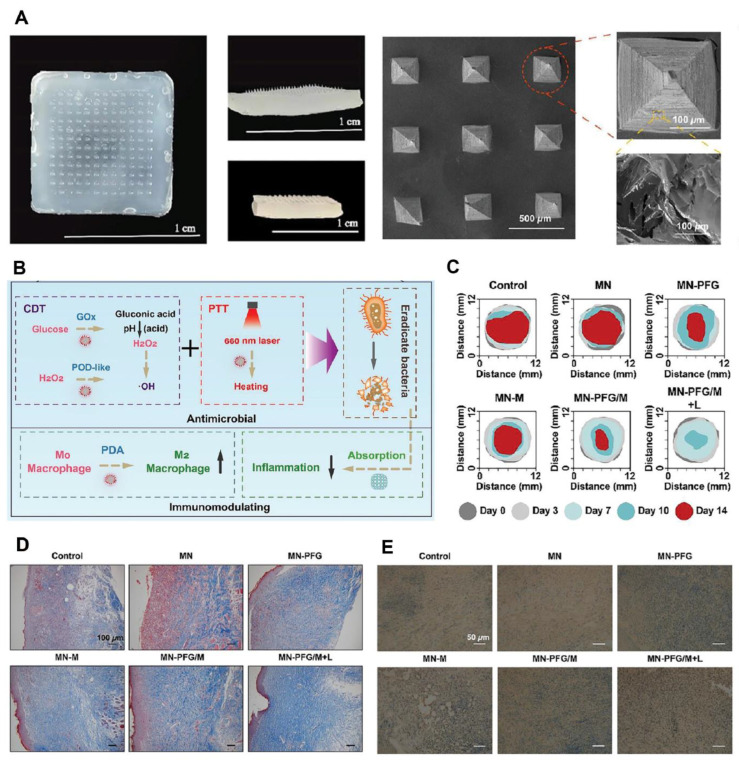
Dissolving microneedles based on CDT for the treatment of skin infection. (**A**) Optical and SEM images of the PFG/M microneedle patch. (**B**) Therapeutic mechanism of PFG/M microneedle for promoting healing of infected wounds. (**C**) Simulation illustrating the wound-healing process. (**D**) Masson staining and (**E**) immunohistochemistry staining of the wound bed. Reproduced with permission from [127] Copyrights 2023, Advanced Science.

Fu et al. [126] developed a nanoreactor that utilized a combination of CDT, PTT, and PDT to exert triple-enhanced antimicrobial effects. Li et al. [127] designed a microneedle patch system with antimicrobial and immunomodulatory properties to promote wound healing (Figure 6). The microneedle encapsulates Fe/PDA@GOx@HA nanoparticles and amino mesoporous silica nanoparticles at the tip and substrate to form PFG/M microneedle nanoparticles. When the tip penetrates the biofilm, Fe/PDA@GOx@HA is released into the wound, where glucose oxidase converts glucose into gluconic acid and produces hydrogen peroxide, which rapidly reacts with iron ions for CDT. Additionally, free polydopamine exhibits excellent photothermal properties under laser irradiation and enhances wound repair by binding to CDT and PTT while promoting M2 macrophage polarization, resulting in synergistic antimicrobial effects. In vivo experiments showed that the PFG/M microneedle light group could kill bacteria, stimulate wound closure, and effectively promote collagen deposition for wound healing.

### 7.2. Antimicrobial Dissolving Microneedles Based on Nanoenzymes

Nanoenzymes are nanomaterials that mimic enzymes. Compared to natural enzymes, nanozymes have unique advantages, such as high stability, low cost, easy modification, and adjustability. In recent years, nanoenzymes have played an important role in the inhibition of bacterial infections as new-generation antimicrobial agents [128,129]. The main mechanisms by which nanoenzymes mediate antimicrobial activity include the modulation of reactive oxygen species (ROS), generation of HOBr/Cl, and scavenging of extracellular DNA [130]. Corresponding to these three antimicrobial mechanisms, the basic design principles for antimicrobial nanoenzymes include the following. (1) Nanoenzymes are used with peroxidase (POD) and oxidase (OXD) activities to catalyze various substrates and generate large amounts of ROS that can target bacterial cell walls, cytoplasmic membranes, organelles, DNA, proteins, polysaccharides, and nucleic acids to kill bacteria. Additionally, nanoenzymes with superoxide dismutase (SOD), catalase (CAT), and other antioxidant enzymes can remove excessive ROS to promote wound healing or reduce inflammatory responses caused by bacterial infection. (2) Nanoenzymes are used with haloperoxidase activity (such as chloroperoxidase, bromoperoxidase, and iodoperoxidase) to catalyze the production of hypohalous acids (HOCl, HOBr, and HOI) from halide ions (Cl^−^, Br^−^, and I^−^) and H_2_O_2_, resulting in irreversible damage to bacteria and biofilms. (3) The hydrolysis of eDNA, a key component of the extracellular matrix, is accelerated using nanoenzymes with DNAase-like activity, in turn eliminating biofilms [128,130].

Antimicrobial nanoenzymes [130] can be categorized into three groups: (1) metal-based nanoenzymes, such as Mxene [131,132], CeO_2_ [133,134], Fe_3_O_4_ [135,136], Cu_5.4_ O [137], CuS [138], MnO_2_ [139,140], MoS_2_ [141], Co_3_O_4_ [142], and V_2_O_5_ [143,144]; (2) carbon-based nanoenzymes including carbon nanotubes [145], carboxylated graphene oxide [146], and graphene quantum dots [147]; and (3) metal–organic skeleton nanoenzymes [148] such as MOF_−2.5 Au-Ce_ nanoenzymes [149] and MOF@COF [150].

**Figure 7:**
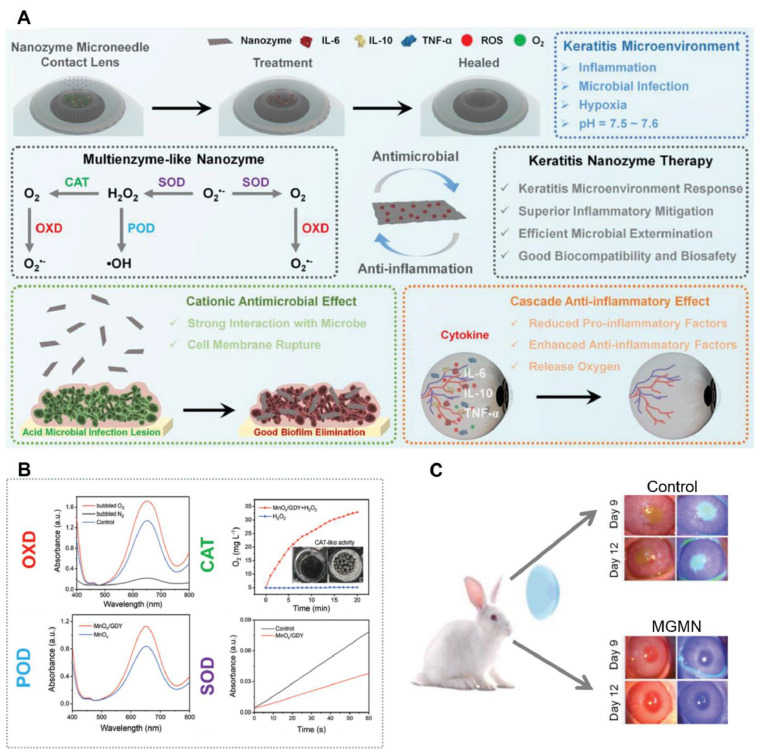
Dissolving microneedles containing nanozyme antibacterial therapy for skin infection. (**A**) Multienzyme-like nanozymes ocular microneedles for Keratitis Treatment. (**B**) Multienzyme-like activities of MnO_X_/GDY. (**C**) Representative photographs of fungal-infected rabbit corneas undergoing various treatment interventions. Reproduced with permission from [151] Copyrights 2024, Small.

Liu et al. [151] designed manganese oxide nanocluster-modified graphene nanosheets (MnO_X_/GDY) as nanoenzymes and loaded them into hyaluronic acid- and polymethylmethacrylate-based ocular microneedles to treat keratitis (Figure 7). The experimental results showed that the microneedles could penetrate the ocular barrier, target the corneal-lesion site through electrostatic interactions, and utilize the biofilm microenvironment at the infection site to activate OXD- and POD-like activities to achieve antimicrobial effects. Simultaneously, SOD and CAT-like activities were exhibited at the site of corneal inflammation to remove excessive ROS, effectively eliminate pathogenic bacteria, prevent biofilm formation, reduce inflammatory responses, alleviate ocular hypoxia, and promote the repair of corneal epithelial damage.

Nanoenzymes have exhibited great advantages and potential in the field of antimicrobials owing to their unique physicochemical properties. However, this is accompanied by biosafety concerns. Although many metal-based nanoenzymes exhibit strong bactericidal properties, metal ions such as Fe^3+^, Zn^2+^, and Cu^2+^ also have strong protein-inactivating effects, making them potentially biotoxic [152]. To alleviate these concerns, researchers have developed various strategies, including reducing the size of nanoenzymes through current modifications and utilizing biodegradable biocompatible molecules, which are effective in reducing toxicity. In addition, establishing a complete safety evaluation system is an indispensable prerequisite for research on nanoenzyme antimicrobials for clinical applications [153].

### 7.3. Antimicrobial Dissolving Microneedles Based on PDT

PDT utilizes exogenous photosensitizers (PSs) to selectively destroy target cells or tissues by generating highly cytotoxic ROS through a series of photochemical reactions with oxygen under light excitation in a specific wavelength range (600–900 nm) [154,155]. In the field of antimicrobials, this method is known as antimicrobial PDT. Antimicrobial PDT has emerged as a promising strategy for combating microbial infections owing to its topical application, broad-spectrum antimicrobial activity, and lack of susceptibility to drug resistance [156].

The main action mechanism of PDT is that under light irradiation, PSs absorb photons and gain energy, transitioning from the ground state to a singlet-excited state. This singlet-excited state is highly unstable and can decay back to the ground state, releasing excess energy such as fluorescence and heat. In addition, it can form a more stable triplet-excited state. From this triplet-excited state, PSs can generate ROS through two pathways: first, by transferring their own electrons or hydrogen atoms to surrounding oxygen molecules, resulting in the production of free superoxide anions (O^2−^), hydroxyl radicals (–OH) and hydrogen peroxide (H_2_O_2_); second, by directly transferring energy to triplet oxygen molecules, leading to the formation of singlet molecular oxygen (^1^O_2_) [154,157].

PSs can be categorized into two classes: organic compounds (such as porphyrins, phthalocyanines, phenothiazines, fullerenes, methylene blue, dihydroporphyrin, and aggregation-induced emission (AIE) luminogens) and inorganic photocatalysts (such as TiO_2_ and ZnO) [158,159,160]. Microbial cells typically have a more negative charge than mammalian cells; therefore, cationic PSs can selectively bind to microbial cells while avoiding binding to host mammalian cells. Porphyrins and their derivatives are the most widely used PSs owing to their strong visible-light absorption and good oxygen quantum yield in the single-linear state. However, the poor water solubility of porphyrin groups in physiological environments results in low ROS yields. To improve the efficacy of porphyrin-based PSs, they are usually loaded onto nanocarriers such as mesoporous silica nanoparticles, supramolecular nanoparticles, metal-organic frameworks, and amphiphilic copolymers [161,162].

**Figure 8:**
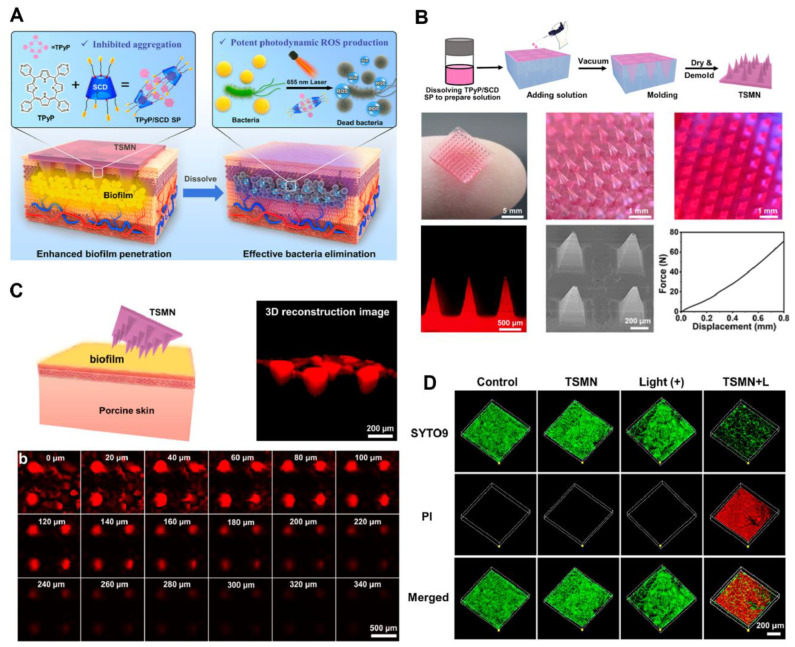
Dissolving microneedles incorporating PDT for the treatment of skin infections. (**A**) Diagram depicting the TSMN for improved penetration and eradication of bacterial biofilms. (**B**) Fabrication and characterization of the TSMN. (**C**) Successful penetration of the *S. aureus* biofilm by TSMN. (**D**) Confocal laser scanning microscopy images depicting representative *S. aureus* biofilms following various treatments. Reproduced with permission from [162] Copyrights 2023, ACS Applied Materials & Interfaces.

The limited ability of PSs to penetrate biofilms limits their effectiveness in eliminating biofilms. Therefore, Wang et al. [162] designed a dissolving microneedle loaded with a supramolecular photosensitizer to enhance the permeability of bacterial biofilms (Figure 8). In this study, Tetra(4-pyridyl)-porphine (TPyP) was encapsulated into the hydrophobic cavity of sulfobutylether-β-cyclodextrin to prepare the supramolecular photosensitizer, which was subsequently dissolved in water to produce the matrix liquid for the microneedle. A dissolving microneedle, TPyP/SCD-dissolving microneedle (TSMN), was then prepared. The TSMN possesses excellent mechanical properties and can easily penetrate the EPS of biofilms, enabling full contact between TPyP and bacteria. The in vivo experiments showed that TSMN effectively eradicated *S. aureus* biofilms with potent photodynamic ROS production with good biosafety.

### 7.4. Antimicrobial Dissolving Microneedles Based on PTT

In recent years, PTT has demonstrated unique advantages in antimicrobial therapy owing to its low invasiveness, deep penetration, high selectivity, and ability to avoid drug-resistant bacteria. PTT utilizes photothermal agents (PAs) to convert light energy into localized heat energy under near-infrared light (650–1350 nm). This process leads to bacterial protein/enzyme denaturation, cell-membrane rupture, evaporation of cytosol, and cell cavitation, thereby achieving efficient sterilization [163].

The application of PAs as photothermal agents is crucial for the success of PTT. Negatively charged PAs on the surface typically exhibit lower delivery efficiency in antimicrobial applications, owing to electrostatic repulsion with negatively charged bacteria. Consequently, various PA modification strategies have been developed. Over the past decade, several types of PAs have been developed for PTT, including noble-metal nanomaterials (e.g., AuNPs and AgNPs), metal sulfides (e.g., CuS and MoS2), inorganic materials (e.g., graphene oxide nanosheets, black phosphorus nanosheets, and Prussian blue nanoparticles), natural materials (e.g., melanin), and other organic compounds (e.g., indocyanine green). Recently, two-dimensional transition metals such as Sc, Ti, Zr, Hf, V, Nb, Ta, Cr, and Mo carbides and nitrides, known as MXenes, have shown potential as photothermal agents owing to their superior photothermal conversion efficiency and good biodegradability [94,164].

In general, the use of PTT alone for antimicrobial treatment is limited because of bacteria’s high resistance to elevated temperatures, and temperatures as high as 70 °C are typically required to completely eradicate them. Elevated tissue temperatures can lead to the formation of microvascular thrombi, increasing the risk of hypoxia and ischemia. Additionally, when tissue temperatures exceed 60 °C, many normal cells are killed. Therefore, photothermal antimicrobial microneedling achieves antimicrobial efficacy with minimal side effects without the need for multiple drugs or therapies [165].

**Figure 9:**
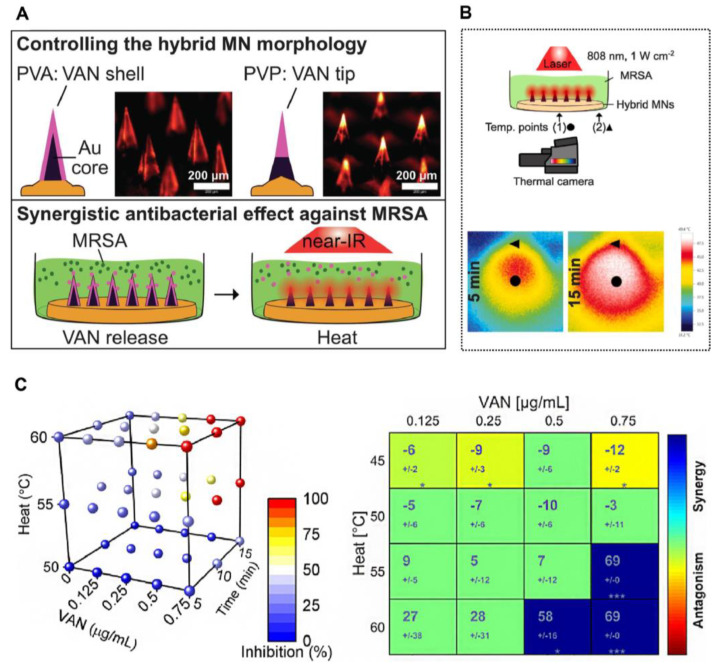
Dissolving microneedles based on PTT for the treatment of skin infection. (**A**) Illustration depicting the antibacterial mechanism of photothermal microneedles. (**B**) Thermal images of the microneedles. (**C**) Assessment of the combined effect of heat and vancomycin on inhibiting the growth of methicillin-resistant *S. aureus* in vitro. Reproduced with permission from [166] Copyrights 2023, Chemical Engineering Journal.

Ziesmer et al. [166] designed a two-layer microneedle array with photothermal antimicrobial effects to treat bacterial skin infections. The microneedle consists of a composite of a water-insoluble near-infrared photothermal core layer and an external water-soluble layer containing vancomycin (VAN) (Figure 9A). When the microneedle delivers the drug to the infected site, the outer matrix layer dissolves to release VAN, producing an initial antibacterial effect. The insoluble photothermal inner layer generates a localized high temperature through the photothermal effect, further enhancing the antibacterial effect. The experimental results showed that after 15 min of near-infrared light irradiation, the local temperature of the microneedle increased to more than 50 °C, exhibiting a significant temperature-elevation phenomenon (Figure 9B). Simultaneously, this localized high-temperature effect and the antibiotic VAN produced a synergistic antibacterial effect (Figure 9C). Compared with photothermal or antibiotic treatment alone, exposure of *S. aureus* to a low concentration (0.25 μg/mL) of VAN solution and heat treatment for 10 min resulted in 48% inhibition, indicating that PTT in the composite microneedle was able to enhance bactericidal effects.

## 8. Antimicrobial Dissolving Microneedles Based on Combination Therapy

Combination therapy is an innovative strategy for treating diseases more effectively by rationally combining multiple therapeutic mechanisms, aiming to increase efficacy, reduce doses, minimize side effects, and slow the development of resistance. This strategy is particularly suitable for overcoming the limitations of monotherapy in antimicrobial therapy. As shown in previous studies, the use of combination therapies can be synergistic, i.e., “1 + 1 > 2”, due to the complementarity of bactericidal mechanisms [167].

**Figure 10:**
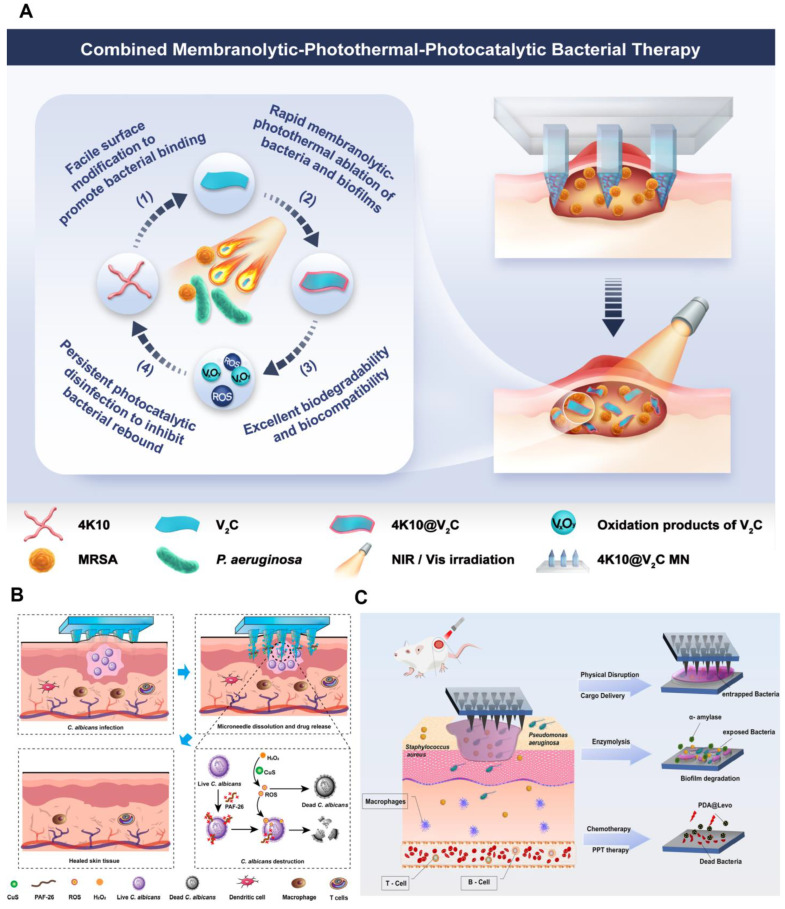
Dissolving microneedles based on combination therapy involving multiple drugs for the treatment of skin infection. (**A**) Combined membranolytic–photothermal–photocatalytic therapy of 4K10@V_2_C microneedle. Reproduced with permission from [168] Copyrights 2023, Chemical Engineering Journal. (**B**) Combination of nanoenzymes and AMPs. Reproduced with permission from [138] Copyrights 2023, Nano Letters. (**C**) Combination of PTT and antibiotics. Reproduced with permission from [172] Copyrights 2022, Chemical Engineering Journal.

Recently, beneficial bacterial membrane-disrupting effects of AMPs have been demonstrated to enhance the efficacy of various antimicrobial therapies. Therefore, they have been suggested not only as broad-spectrum antimicrobial agents but also as highly effective sensitizing adjuvants [93,100,168,169,170]. This is because AMPs increase the permeability of bacterial–cell membranes, facilitating the aggregation of antimicrobial drugs into the bacterial cell and weakening the ability of bacteria to resist external stimuli. Feng et al. [168] constructed vanadium-based MXene composite nanosheets 4K10@V_2_C by electrostatically coating a four-armed host-defense peptide, 4K10, onto the surface of V_2_C MXene nanosheets. Then, dissolving microneedles were prepared by a multistep centrifugation method (Figure 10A). The system integrates membranolytic, photothermal, and photocatalytic capabilities to realize effective photo-excited bacterial killing through a microneedle-assisted approach. In vivo experiments showed that the microneedle therapy was superior to commercially available broad-spectrum antimicrobial agents without significant in vivo toxicity. A combination of multiple therapies exhibited better antibacterial and anti-biofilm activities than PTT alone, achieving rapid localized biofilm lysis, photothermal bacterial ablation, and sustained photocatalytic bactericidal action of V_x_O_y_.

Lei et al. [171] designed a dissolving microneedle loaded with bacteria-responsive composite nanoparticles (Ce6@GNP-Van). Composite nanoparticles can specifically target *S. aureus*, degrade Ce6 in situ by gelatinase secreted by the bacteria in the infected area, and release Ce6, which kills the bacteria through the combined effects of antibiotics and PDT under laser irradiation. The in vivo experiments showed that the microneedles completely dissolved after 10 min of skin zapping. When the Ce6@GNP-Van dissolving microneedles were applied to diabetic mouse wounds infected with *S. aureus* biofilms, the wounds healed completely within 15 days. Microneedles achieve efficient biofilm removal and cascade activation during wound healing.

Deep fungal infections pose a major challenge to public health because of the difficulties in their treatment and recurrence. Wang et al. [138] developed a novel biodegradable microneedle patch (CuS/PAF-26 MN) to treat deep fungal infections. The microneedle comprises hyaluronic acid and sodium carboxymethylcellulose and contains internal antifungally active copper sulfide nanoenzymes (CuS NEs) and AMPs (PAF-26) (Figure 10B). When the microneedle is inserted into the skin, the released CuS NEs catalyze the generation of highly toxic ROS from hydrogen peroxide. Simultaneously, the AMP, PAF-26, exerts a membrane-breaking effect, allowing the rapid action of ROS on the fungus and achieving a more efficient antifungal effect. In vivo efficacy showed that nodules treated with CuS, PAF-26, or CuS/PAF-26 microneedles could be reduced to 48%, 65%, and 19% of their initial size, respectively. After 10 days of treatment, the number of fungal residues in the CuS/PAF-26 microneedle group was essentially equivalent to that in uninfected skin, suggesting that combining nanoenzymes and AMPs is more effective than using either component alone.

Biofilms are a major obstacle in the treatment of bacterial infections because they can significantly impede drug penetration, thereby reducing therapeutic efficacy. To overcome this challenge, several drugs with biofilm-disrupting effects have been developed to enhance the efficacy of other drugs. Yu et al. [172] designed a dissolving microneedle that enzymatically disrupts bacterial biofilms using α-amylase to break down the EPS, which subsequently kills bacteria through a combination of PTT and antibiotics (Figure 10C). In vivo experiments demonstrate that the microneedle eliminates biofilms from infected wounds and eradicates exposed bacteria. This treatment significantly reduces the inflammatory response and promotes wound healing. Importantly, gentle PTT does not harm normal tissues, indicating a favorable safety profile.

Infected wound healing is a dynamic process involving the timely transition through organized phases. However, infected wounds often experience prolonged inflammation due to microbial overload. Microneedles provide a potential platform for antibacterial action and promote tissue regeneration, which is essential for restoring microbial homeostasis. Qi et al. [173] designed a core-shell microneedle named CSMN@TA-Mg/LDEV to promote wound healing by the sequential delivery of tannic acid–magnesium (TA-Mg) complexes and extracellular vesicles from *Lactobacillus druckerii* (LDEVs). The TA-Mg released from the outer shell exhibits excellent antibacterial properties and ROS scavenging activity, aiding the transition to the proliferative phase. Subsequently, LDEVs released from the core are used to promote wound healing and regulate microflora, thus restoring microbial homeostasis of the skin. Their research indicates that CSMN@TA-Mg/LDEV not only inhibits pathogenic bacteria but also significantly increases microbial diversity, leading to accelerated wound healing and improved appearance and function of the healed skin.

The iontophoresis technique is a non-invasive drug delivery technique that employs a mild electric current to drive drug penetration through the skin barrier by electromigration and electroosmosis, thus overcoming limitations associated with traditional transdermal drug delivery methods. This method not only improves drug penetration rates but also allows for precise control over drug administration [174]. The efficiency of drug delivery can be further enhanced by integrating iontophoresis with dissolving microneedle technology. Zafar et al. [175] developed a strategy to enhance the activity of linezolid in combating oral biofilms by using chitosan microneedle CSHP3 with iontophoretic control. When combined with the iontophoresis technique, the microneedle increases the concentration of linezolid in the biofilm by facilitating its penetration through an electric field. In vivo anti-biofilm studies showed that *S. aureus* biofilms from oral mucosal wounds in rabbits treated with CSHP3-iontophoresis combination were undetectable as pathogens by day 7, and the mucosal tissue showed signs of regeneration. This novel microneedle system, when paired with iontophoresis, effectively inhibits the formation and growth of oral biofilms, offering a promising new strategy for the treatment of oral infections.

## 9. Conclusions, Clinical Challenges, and Perspective

SSTIs should be carefully addressed as they can rapidly deteriorate, leading to serious local complications or systemic infections, such as sepsis. The difficulty in treating these infections lies in the fact that they may be caused by multidrug-resistant microorganisms capable of forming impermeable biofilms that reduce the efficacy of drugs. Additionally, effective drug delivery may be challenging because of the involvement of deep tissues at the infection site.

Compared with conventional topical formulations, dissolving microneedle patches offer a highly effective solution that promotes drug penetration through the stratum corneum barrier and increases drug concentration at the site of infection. This type of topical drug delivery not only improves efficiency and safety but also enhances patient compliance. Currently, microneedles loaded with antibiotics have demonstrated significant potential for various applications. The successful development of microneedles based on QSIs, AMPs, and antimicrobial-matrix materials has provided a theoretical and practical foundation for advancing the clinical translation of antimicrobial microneedles. Additionally, some new antimicrobial therapies (e.g., CDT, nanoenzyme antimicrobial therapy, PDT, and PTT) not only perform well when used individually but also exhibit excellent antimicrobial efficacy when combined, offering a novel approach to overcome drug resistance and improve therapeutic effectiveness.

Over the past two decades, dissolving microneedles have seen substantial progress in therapeutic diagnostics and various other fields. However, antimicrobial dissolving microneedles are still in the pre-developmental stage compared to traditional drug delivery systems [176]. The clinical application of dissolving microneedles for antimicrobial drug delivery to achieve safe and effective therapy faces several challenges. Firstly, enhancing the drug loading capacity within the microneedles is essential to ensure adequate delivery of antimicrobials to the infection site. The release rate and dissolution characteristics of the antimicrobials must also be precisely controlled to facilitate sustained release and improve therapeutic efficacy. Safety assessments, including the biocompatibility and long-term safety of the microneedle materials, are critical. Moreover, establishing clear metrics is vital for determining the success of drug delivery. Lastly, the industrial production of dissolving microneedles requires sterile conditions, presenting numerous challenges that must be addressed to enable mass production.

More specifically, among the various existing antimicrobial microneedle systems, dissolving microneedles loaded with conventional antibiotics is currently dominant in terms of druggability. This is because these antibiotics have been clinically proven over a long period, with clear efficacy and safety data. Their pharmacokinetic and pharmacodynamic properties have been extensively studied, and well-established processes exist for manufacturing, stability, and regulatory approval [177]. In contrast, other types of antimicrobial microneedles require additional studies to validate their safety, stability, and manufacturing feasibility despite their attractiveness in terms of innovativeness and potential efficacy. For example, while QSI and AMPs offer novel mechanisms of action, their production costs and scale-up production capabilities are yet to be validated [178]. Furthermore, CDT and nanoenzyme antimicrobial therapies require further investigation regarding long-term biocompatibility and potential toxicity, and PDT and PTT depend on specific devices and technologies that may limit their widespread use.

In addition to treating localized skin infections, dissolving microneedles have shown potential for the treatment of systemic infections. Although this is not their primary application, dissolving microneedles offers a novel and patient-compliant route for drug delivery. Systemic antimicrobial efficacy may be enhanced through specific drug carriers and microneedle design by facilitating the direct passage of drugs through the skin into circulation or by stimulating an immune response. However, challenges also exist, including ensuring the systemic bioavailability and stability of the drug, designing appropriate drug-release kinetics to maintain effective blood concentrations, and assessing and minimizing possible systemic side effects [179]. The homogeneity of drug distribution in the body must also be addressed to ensure that the drug can effectively reach the site of infection and overcome the barriers faced during regulatory approval and clinical application.

Therefore, to effectively explore clinical trials with dissolving microneedles, it is essential to establish well-defined objectives that focus on evaluating drug efficacy and absorption. These objectives are the foundation for selecting suitable participants, which is paramount for ensuring the safety and efficacy of the trial. The selection process must also consider the determination of the optimal dosage, which is critical for striking a balance between therapeutic potency and patient safety. Safety must be the foremost consideration, not only in participant selection but also in guiding the choice of materials designed to reduce the potential for adverse reactions. Throughout the trial, the effectiveness of the microneedle technique must be meticulously measured, including the monitoring of drug distribution patterns. Furthermore, comprehensive data collection and meticulous analysis are indispensable for the precise evaluation of the trial’s results.

With advancements in materials science, biotechnology, and nanotechnology, antimicrobial microneedle technology is expected to enhance the precision and efficacy of drug-delivery systems. By improving the design and materials of microneedles to enhance drug stability and bioavailability, antimicrobial microneedles are anticipated to become a significant treatment option for SSTIs and have the potential for broader applications in healthcare, offering patients safer, more effective, and more convenient treatment options.

## Figures and Tables

**Table 1:** Comparison of various types of microneedles.

Type of Microneedles	Materials Used for Fabrication	Preparation Methods
Solid microneedles	Silicon, lithographic epoxy, polycarbonate, polymethylmethacrylate, polylactic acid and polyglycolic acid, stainless steel, titanium, nickel, ceramics	Laser cutting, chemical etching
Hollow microneedles	Silicon, metal, glass, polymers, ceramics	Microelectromechanical systems, ion etching, wet chemical etching, deep x-ray lithography, integrated photolithography molding, 3d printing
Coated microneedles	Stainless steel, polymer	Spraying method, inkjet printing, dip-coating method
Hydrogel microneedles	Poly(methyl vinyl ether-alt-maleic acid) polymers, poly(methyl vinyl ether-*co*-maleic anhydride), hyaluronic acid methacrylate, polyvinyl alcohol	Solvent casting, stretch lithography, droplet-blowing laser processing, thermocompression micro-injection molding, and ultrasonic welding
Dissolving microneedles	Sodium hyaluronate, chondroitin sulfate, carboxymethyl cellulose, sodium alginate, dextran, maltose, polyvinylpyrrolidone, poly(vinylpyrrolidone-*co*-methacrylic acid), polymers including polylactic acid, polyglycolic acid, and poly(vinylpyrrolidone-*co*-methacrylic acid)	Solvent casting, stretch lithography, droplet-blowing laser processing, thermocompression micro-injection molding, and ultrasonic welding

**Table 2:** Representative dissolving microneedles in clinical trial stage.

Clinical Trial Titles	Applications	Active Ingredients	Sponsors
Microneedle patch for psoriatic plaques	Psoriatic plaques	HA	The Catholic University of Korea
Microneedle array plus doxorubicin in cutaneous squamous cell cancer	Cutaneous squamous cell cancer	Doxorubicin	University of Pittsburgh
Soluble hyaluronic acid microneedle vs. Non-ablative fractional laser on infraorbital wrinkles	Infraorbital wrinkles	HA	Shanghai Dermatology Hospital
Transdermal microneedle lignocaine delivery versus emla patch for topical analgesia before venipuncture procedure to adults in a clinical setting	Topical analgesia	EMLA 5% patch	Universiti Kebangsaan Malaysia Medical Centre
Efficacy of transdermal microneedle patch for topical anesthesia enhancement in pediatric thalassemia patients	Topical anesthesia enhancement	1 finger-tip unit EMLA	Universiti Kebangsaan Malaysia Medical Centre
Measles and rubella vaccine microneedle patch phase 1–2 age de-escalation trial	Measles and rubella vaccine	Measles rubella vaccine (MRV-SC)	Micron Biomedical, Inc. (Atlanta, GA, USA)
Placebo microneedles in healthy volunteers (Part I) and efficacy/safety of doxorubicin microneedles in basal cell cancer subjects (Part II)	Efficacy/safety subjects	Placebo-containing MNA	SkinJect, Inc. (Pittsburgh, PA, USA)
Dexmedetomidine hydrochloride microneedle patch	Preoperative shock in children	Dexmedetomidine hydrochloride	Guangzhou Novaken Pharmaceutical Co., Ltd. (Guangzhou, China)
